# Supplementary material for: Predictors of early initiation of breastfeeding in Indonesia: A population-based cross-sectional survey
Source: PLoS One. 2020 Sep 24;15(9):e0239446. doi: 10.1371/journal.pone.0239446 (PMC7514028; doi:10.1371/journal.pone.0239446)
Supplement: Attachment — Submitted filename: Response to Reviewers.docx [file pone.0239446.s001.docx]

*Review Comments to the Author

Please use the space provided to explain your answers to the questions above. You may also include additional comments for the author, including concerns about dual publication, research ethics, or publication ethics. (Please upload your review as an attachment if it exceeds 20,000 characters)*

**Reviewer #1:** *The manuscript submitted by Maria Gayatri and colleagues aims to investigate predictors of early initiation of breastfeeding in Indonesia. Overall, this manuscript presents results that would be of interest to the community of scientists and clinicians concerned with this problem. Prior to publication, the following points should be addressed.

Overall: I recommend editing the text to achieve a more appropriate scientific language. Furthermore, the quality of writing should be verified, since punctuation marks issues can be identified over the manuscript, compromising the structure and demanding extra attention to interpret the information.*

Response: We have reformatted the manuscript to a more appropriate scientific language.

*Abstract:
-Introduction and methods repeat the objective of the study. Please, mention it just once.
-You mentioned “EIBF”, but this term was fully described later.
-Please, add the confidence Interval for EIBF proportion.*

Response: Thank you. We have added the confidence interval for EIBF proportion. We have revised the abstract and only mentioned the objective just once.

*Introduction:
-The introduction is very succinct. Authors could add information about the factors that most commonly affect EIBF and enrich the text with data on the global epidemiology of EIBF, since these are the objectives of the study (% of EIBF and predictors).
-You can start using “EIBF” abbreviation in this section.*

Response: Thank you for your comments. We have added data on global epidemiology of EIBF and discussed the factors influencing EIBF. We have started using the acronym EIBF in this section after mentioning early Initiation of Breastfeeding with expanded initials at the beginning of the Introduction. We have also mentioned The Ten Step to Successful Breastfeeding from WHO/UNICEF with references.  *Methods:
Dependent variable:
-For definition of EIBF you can also cite the “Ten Steps to Successful Breastfeeding” of WHO.*

Response: Thank you. We have added the definition of EIBF based on the WHO/UNICEF initiative - Then Steps to Successful Breastfeeding.

*-I recommend you reorganize the text as follow: mention the dependent variable, its definition and finally how it was measured and categorized in the analysis.*

Response: For the dependent variable we have added and reorganised as suggested by the reviewer.

*Independent variable:
-The wealth index of the woman’s family is not a medical characteristic. Please, explain how this variable was constructed and what it was based on.*

Response: Thank you for your suggestion. We have described Wealth Index as a household level variable. This variable was constructed by The Demographic and Health Survey team, but we have added the details of how this index was constricted with adequate references.

*-The text is not well organized. The paragraph between line 100 to 106 - in page 4, is repeated in the text*

Response: Thank you. We have deleted the repeated text in the methods section.

*-The levels of the independent variable are not clear. Please, reorganize the text explaining the levels, the variables included in each level, and how they were categorized for analyses.*

Response: Thank you for pointing this out. The method has been corrected and organized according to individual level, household level and community level variables. The article has been corrected on line 120-162 (check the line in the final manuscript).

*-The following variables need more details for better understanding: type of assistance during delivery and child’s size at birth (in lieu of birth weight).*

Response: We have added in the methods section the details of type of assistance during delivery and child’s size at birth.

*Methods of Analysis:
-I cannot comment on complex samples design statistical method as I am not familiar with it.
-Please, add the abbreviation of odds ratio in brackets.*

Response: Thank you. We added the abbreviation of adds ratio in the bracket.

*-Bernoulli's formula was presented but not mentioned in the text as “formula (1)”.*

Response: Noted. We have added “formula (1)” in the text. Thank you.

*-what program was used to perform the statistical analyses?*

Response: We have used Stata version 15 (StataCorp, College Station, TX). We have added the statistics tools in the text.

*-what does “(n.d.)” mean? Page 6, line 145.*

Response:” n.d” stands for “not dated”. This is a standard notation in citing a reference for which the date or year of publication is not available. However, we have deleted “n.d.” in the text.

*Results:
-You mentioned “early initiation of breastfeeding (EIBF)” before, so use the acronym from then on. Make sure you align this detail throughout all the text.*

Response: Thank you. We have revised the paper accordingly and by mentioning Early Initiation of Breastfeeding with expanded initials at the beginning and used the acronym EIBF in the remainder of the text.  *-The outcome of interest was previously defined. Please, do not repeat the information in this section.*

Response: Thank you, we have deleted the definition of outcome in the result because it is already mentioned in the methods section.

*-What table was the following affirmation based on? “The distributions of early initiators of breastfeeding….with respect to each of the predictor variables appear not to be dissimilar from one another…” Table 1 does not allow this affirmation.*

Response: Thank you. We have deleted this sentence since it does not have much bearing on the results of the analysis.

*Table 1:
-Mother's age at childbirth has n=6.615, please mention in footnotes if there are any missing data.
-please, note that some variables have n=6.617 instead of the referred 6.616 (Birth order of the child, Skin-to-skin contact, etc.). Check all variables and inform the correct n.*

Response: Thank you. There are no missing data. We have corrected the error. Now the *n* is 6,617 throughout. However, we have removed Table 1 because it is very nearly similar to Table 2. Now there is only table showing the distribution of respondents and their characteristics.

*-information on “education and working status of the parents” is confused. Please, review the writing (line 182 to 191). For instance, with respect to the following sentence: “However, the opposite appears to be true with respect to father’s education, where more children of nonworking fathers experience delayed initiation of breastfeeding.”, are you talking about father’s education or working status?*

Response: Thank you. We are referring to father’s occupation. We have revised the text to make this clear.

*-With respect to the following sentences: “Perhaps children whose fathers are not working have working mothers…”, “….it should be because the importance of early initiation of breastfeeding must be conveyed to the pregnant women during their ANC visits”, “C-section deliveries may have higher levels of education as found elsewhere [19],” This kind of comments/suggestions is appropriate in the discussion section. Please, try to be more objective in the result section by presenting only the interpretation of the results shown in tables. See the following example: (mother living with her partner and having one or more children were positively associated with a higher probability of EBF. Conversely, mother aged <19 years and using pacifier, were related to a lower probability of EBF. However, there were no significant associations between EBF and other variables, including maternal education, self-reported skin color and household wealth index).*

Response: We have moved these comments to the discussion section. Regarding the example you have given, may we know its source?

*-About “Place of delivery (home versus health facility)”, should be described in the methods section.*

Response: We have added the variable of place of delivery in the methods section.

*-Line 210, “…as indicated by the χ2 and p-values”, the statistical analysis was described before. Avoid repeating information in the text.*

Response: Thank you. We have deleted the repetition.

*-“(Table 2) in line 210”. You mentioned before that you were talking about this table.
Title: Predictors of Early Initiation Breastfeeding or Predictors of Early Initiation of Breastfeeding?*

Response: The previous Table 2 has changed to be Table 1 in the revised manuscript. We have revised the title of the (new) Table 1 into “Characteristics of women having their most recent birth in the two years preceding the survey according to initiation of breastfeeding status, Indonesia 2017”. ”

*-The first paragraph after this title is not necessary (line 221 to 234). You have already defined the outcome of interest, explained the statistical analysis and described the factors associated in the bivariate analyses.*

Response: Agreed. We have removed the statements.

*-Details on “father’s work (or occupation of the woman’s husband)” categorization (in agricultural and non-agricultural activities) was not given in methods.*

Response: We have added it in the methods section.

*-Is the fact father works in agricultural activities related to EIBF? (OR 1.85 (0.94, 3.65). Please, review the interpretation of the results.*

Response: We have revised the article. There is no difference in the practicing of EIBF between children whose fathers are not working or working in agriculture. However, children whose fathers are working in non-agriculture activities are about twice as likely to have an EIBF compared to children whose fathers are not working.

*-Table 3 was also calculated by the authors from 2017 IDHS data?*

Response: Yes, table 3 was calculated by the authors from 2017 IDHS data. In the revised manuscript, Table 3 becomes Table 2. Thank you.

*-Table 3 does not need to show the p-value.*

Response: We thank the reviewer for this comments. However, we believe that p-value is important to conclude the analysis. Therefore, we have retained the p-values in Table 2.

*-I recommend presenting the results in the same order in which the variables were listed in the tables (according to each level of variables).*

Response: We appreciate the reviewer’s comment and agree that presenting the results in the same order is important. We have revised it for improvement to our paper.  *Discussion
-EIBF prevalence is higher than that in the Philippines (56.9%)?*

Response: Thank you for pointing this out. EIBF prevalence in Indonesia is 56.6% and it is almost equal to that in the Philippines (56.9%). Both round up to 57%.

*-If “health providers and health facilities are mandated with promoting the initiation of breastfeeding within one hour of the birth of a child”, why is the prevalence of EIBF in Indonesia still lower than some other countries? According to WHO, a country is considered “very Good” when at least 90% of the children initiate BF within one hour of life. Indonesia would be considered “Good”. I recommend you cite and discuss these ratings. (WHO reference: Infant and Young Child Feeding. A tool for assessing national practices, policies and programmes. Geneva. 2003)*

Response: Thank you for pointing out this useful source. We have added the WHO reference that based on the guidelines for writing, the practice of EIBF in Indonesia is categorized as good (50-85%).

*-About skilled birth attendants: “The results of the present study are similar to those of other studies”, where were these studies carried out? in what year were they performed? please give more details of the studies you are comparing your results with.*

Response: We have added the studies and the year to the manuscript then compared with our results.

*-Please compare the results of "size at birth" with other studies. This result has not been widely discussed.*

Response: We have added baby’s size at birth to the discussion and put the justification based on other studies about EIBF.

*-(line 332) “mothers from middle households had lower odds of EIBF compared to those from poorer households”, but it was not significant, so why are you mentioning this category?*

Response: Agreed. We have removed it. *-(line 334) “women living in households with middle and rich wealth index are supposed to be more educated and more knowledgeable about the benefits of EIBF”. Please, cite a reference for this affirmation. Please, compare the results with other studies besides reference 28.*

Response: We have provided evidence that women living in households with middle and rich wealth index are more educated and that higher education promotes better knowledge about breastfeeding and health.

*- Mother working status result has not been widely discussed.*

Response: We have added related to mother working status under the discussion of wealthier households.

*-I recommend discussing the results in the same order as they were presented in the results section.*

Response: Thank you. We have revised the discussion in the same order as in the methods and results section.

**Reviewer #2:** *This study examines the predictors of early initiation of breastfeeding among Indonesian women. It stands out for the use of a large and nationally representative sample; however, certain considerations need to be made:*

*The paper could be written more concisely. Some parts are repetitive and confusing (for example, lines 98-109) and some sentences are unnecessary (for example, lines 221-228).*

Response: We have revised the paper and removed the repetitions as far as possible.

*I recommend that the abstract should be rewritten to make it clearer and objective.*

Response: We have revised the abstract.

*In the methods section, authors should include more information about data collection (for example, how was the questionnaire applied?) and rewrite the description of covariates to make it clearer and easier to follow.*

Response: Thank you for your comments. We have re-written the methods in the same order as that of the order of analysis to clearer and easier to understand. We have also described the data collection in more detail.

*The results section should contain only results, no discussion.*

Response: Thank you. We have now included only the results here and moved the rest to the Discussion section.

*This section can be more concise as well. Table 1 and 2 are very similar, so the authors should consider presenting a combined table.*

Response: Thank you for your suggestion, we have removed Table 1 and kept Table 2 (which has now become Table 1). In the revised manuscript, we only use (the new) Table 1 to show the characteristics of the respondents according to early breastfeeding status. **.**

*Lastly, in the conclusion section, I suggest including a summary of the main findings of this study (which are the main predictors associated with the outcome).*

Response: Thank you. We have added a summary of the main findings.

*I do have some minor comments below:
1. Line 145: Please check the information inside the parentheses.*

Response: Thank you. We have removed the information inside the parentheses. (n.d.) means not dated, i.e. the date or year of publication of the source is not known.

*2. Line 146: What does ICF mean?*

Response: According to Wikipedia (<https://en.wikipedia.org/wiki/ICF_International>):

“ICF was founded in 1969 as the Inner City Fund. …. The company was reorganised as a consulting firm and renamed itself ICF Incorporated. In 2006, the consultancy was renamed ICF International….” .

However, in all publications and references it is cited as ICF International, which provides technical assistance to all Demographic and Health Surveys (DHS) Program, which is funded by the U.S. Agency for International Development (USAID). ICF International assisted in the preparation of the 2017 Indonesia Demographic and Health Survey report writing.

*3. Line: The title of Table 1 can be improved.*
Response: Thank you. We have deleted Table 1 because it is very nearly similar to Table 2.

**Reviewer #3:** *Overall comment: This is a paper exploring the predictors of early initiation of breastfeeding using DHS data from Indonesia. This is a topic with a strong body of evidence with data report from multiple countries. The paper has merit and brings reliable analysis, however, the introduction needs to be reviewed including detail about the context (Indonesia) as well as more detail should be provided in the methods section and extensive revisions in reporting results are needed. Below I provide a detail comment and suggestions for the authors to improve the manuscript.*

Response: We have revised the Introduction, and the methods and results sections to address your comments. *ABSTRACT
Lines 13-15. Please clearly define your research question and its importance. The introduction section can be expanded to briefly contextualize why it is important to understand the predictors of early breastfeeding initiation in general and why to study this problem is important to the given population.*

Response: Thank you. We have revised the Abstract to address your comment.

*Lines 16-24. In the method section, the first sentence can be removed as it repeats the objective of the study already stated.*

Response: Thank you. We have removed the first sentence.

*Lines 24-28. Revise the report of results including OR and comparison groups when appropriate.*

Response: We have added the OR and comparison groups in the results.

*Line 29-32. Revise the conclusion to reply to your research question(s). If the focus of the study is breastfeeding within the first hour why its conclusion is addressing breastfeeding for up to years, please keep your conclusion limited to what your data can support.*

Response: We have revised the Conclusion to address your comment.

*INTRODUCTION
Overall comment. I would recommend expanding the introduction to state the problem beyond the benefits of breastfeeding. The authors should consider including one or two paragraphs in the introduction demonstrating what has been identified in prior studies and what are the gaps. Why is important to identify predictors of early breastfeeding initiation? What is this studying adding to the literature or to the context? Early breastfeeding initiation is influenced by several modifiable factors - there is a body of evidence showing factors associated with this practice and none were mentioned. Also, early breastfeeding initiation is highly linked with hospital practices, for instance, this is a practice endorsed and part of the Baby-Friendly Hospital Initiative which was not mentioned in the introduction.*

*Line 57-58. Please clarify what means “losing their child in the neonatal period”. Is this related to increased neonatal mortality? If so, provide the rationale and more evidence on this statement*

Response: Losing a child in the neonatal period means the death of the child in the neonatal period. Sources supporting this statement have been cited in the sentence. The rationale why a child might die in the neonatal period when breastfeeding is initiated late is that the child does not receive “the highly nutritious maternal colostrum that reduces the risk of microbial translocation, accelerates intestinal maturation and promotes resistance to infection [5–9]”. This is stated in the previous sentences.  *METHODS
Overall comment:
The methodological steps described in this section does not match with the results presented. Please revise the methodological steps: 1) descriptive analysis, 2) bivariate analysis - indicating which statistics were used (chi-square or logistic regression, not both), 3) multivariate analysis (predictors of EIBF) and make clear the goal of each of methodological steps. For each step, it is expected a matching table displacing results.*

Response: The results are presented in the order suggested by the reviewer. There are only two tables included in the revised manuscript. We have removed the chi-square results and used only the bivariate logistic regression for bivariate analysis. Table 2 refers respectively to bivariate analysis and logistic regression (multivariate) analysis and they have been referred to each time the results of a bivariate or multivariate analysis are discussed.

*The authors conducted a bivariate analysis to select predictors of breastfeeding early initiation and this name should be used throughout instead of “simple” logistic regression. Please mention the level of significance that what was considered in the bivariate analysis to select variables to the multivariate analysis. Also, please clarify why was used a chi-square test and bivariate logistic regression and how both analyses informed the selection of independent variables. Please provide a rationale to use both methods since this is not clear in the methods or results section.*

Response: This has been done and stated at the appropriate places of the manuscript. *Please review the English verbal tense throughout. A few parts of the methodology are in present and others present perfect, please choose one and be consistent throughout.*

Response: We have red and re-read the manuscript to remove any discrepancy in tense.

*Minor comments:
Line 84-91. Please clarify if mothers who did not breastfeed were included or excluded.*

Response: The mothers who did not breastfeed were excluded from the analysis. This is stated in the description of the sample, namely “which comprise a weighted sample of 6,616 women who had their most recent births in the preceding two years and who ever breastfed their babies”.

*Line 92-109. Why were these predictors chosen? Was this based on literature review or data availability? What is the plausibility of selecting these predictors? Was there any framework used to select variables?*

Response: The predictors are chosen based on literature review and data availability. This study is based on secondary analysis of the 2017 Indonesia Demographic and Health Survey, so the choice of the predictors is also based on the data availability.

*Line 110. Remove the word “methods”*

Response: Thank you. We have removed it.

*Line 111. Remove the word “basically”*

Response: Thank you. We have removed it.

*Line 114 -115. Remove the word “simple” on both lines*

Response: Thank you. We have removed it.

*Line 115. Remove the word “simple” logistic regression.*

Response: Thank you. We have removed it.

*Results
Overall comment:
The result section is long and repetitive. It has to be streamlined with the description of methodological steps. Tables 1 or 2 should be removed or revised to show a descriptive analysis of the whole population (not per outcome) and bivariate analysis. In the text, authors should focus their report in the most important results not show interpretations, interpretations should be placed in the discussion section. For instance, the authors could highlight important characteristics of the population studied and which were the variables selected in the bivariate to the multivariate analysis.*

*Line 160 - Remove the word the definition for early breastfeeding initiation. Once this was defined in the method section there is no need for repetition. Also, if authors will opt for using an acronym for early initiation of breastfeeding (EIBF), please define it for the first time in the text and use this throughout.*

Response: Thank you. We removed the definition of EIBF since it had already mentioned before. We also have revised the manuscript and used the acronym of EIBF after it is describe clearly in the introduction.

*Tables 1 and 2. Please clarify and justify the need for having these two tables. I strongly recommend removing table 1 and keep with table 2.*

Response: Thank you for your suggestion. We have removed Table 1 and kept Tables 2 and 3 of the previous draft, which now become Tables 1 and 2 in the revised draft.

*Line 156. Please remove subheading “Characteristics of respondents”*

Response: Thank you. We have removed it.

*Line 220. Please remove subheading “Predictors of Early Initiation Breastfeeding”*

Response: Thank you. We have removed it.

*Lines 161-210. Please revise accordingly overall comment above.*

Response: Thank you. We have revised it.

*Lines 221-228. Consider summarizing it and moving it to the analysis section.*

Response: We have revised the paper, and put in the analysis section.

*Lines 235-261. Please summarize and revise accordingly the overall comment above.*

Response: Thank you for your suggestion. We have revised the results in the same order with the methods and the discussions.

*Table 3 is confusing, please make sure it is necessary to present bivariate analysis results in this table. Again, consider choosing between chi-square and bivariate logistic regression to select variables to multivariate model, detail this step in the methods, and provide a clear cut-off point to include or not a variable in the model.*

Response: Thank you for your suggestion. We now use bivariate logistic regression to select the variables as predictors in the multivariate model. Table 2 presents the results of simple and multivariate logistic regression analysis with unadjusted and adjusted odds ratios. We have mentioned clearly in the methods that “The final model includes explanatory variables for which the level of significance is less than or equal to 5 percent (P ≤ 0.05). Odds ratios (OR) are reported with a 95% confidence interval (95% CI).” *Minor comment: please make sure tables follow the same formatting style.*

Response: We have tried to make the formatting of the tables as similar as possible to one another. *Discussion:
Overall comment: Consider moving a few sentences from Indonesia context to the introduction, this will help readers to understand the context of the study earlier. Also, was there any hypothesis the authors were trying to explore? Or was this an exploratory study? What gaps this analysis fills?*

Response: This study is further analysis of cross-sectional research. This research will answer the research question of “what is the strongest predictors of EIBF”. The result will be useful to strengthen the policy related to EIBF.

*Lines 285-288/298-300/310-312. Do not report results in the discussion.*

Response: Thank you. We have revised the paper.

*Line 316 – this sentence seems to be incomplete.*

Response: Thank you. We have revised the sentences in the revised paper. It is in line 343.

*Conclusion: The conclusion lacks to respond to the research question. Why is important to know the predictors of EIBF?*

Response: It is important to understand the predictors of EIBF so that appropriate interventions can be designed to strengthen help increase the prevalence breastfeeding for newborn babies. The Conclusion section of the paper has been modified accordingly.

*6. PLOS authors have the option to publish the peer review history of their article (*[*what does this mean?*](https://journals.plos.org/plosone/s/editorial-and-peer-review-process#loc-peer-review-history)*). If published, this will include your full peer review and any attached files.


If you choose “no”, your identity will remain anonymous but your review may still be made public.


Do you want your identity to be public for this peer review? For information about this choice, including consent withdrawal, please see our* [*Privacy Policy*](https://www.plos.org/privacy-policy)*.

Reviewer #1: No

Reviewer #2: No

Reviewer #3: No


[NOTE: If reviewer comments were submitted as an attachment file, they will be attached to this email and accessible via the submission site. Please log into your account, locate the manuscript record, and check for the action link "View Attachments". If this link does not appear, there are no attachment files to be viewed.]


While revising your submission, please upload your figure files to the Preflight Analysis and Conversion Engine (PACE) digital diagnostic tool,* [*https://pacev2.apexcovantage.com/*](https://pacev2.apexcovantage.com/)*. PACE helps ensure that figures meet PLOS requirements. To use PACE, you must first register as a user. Registration is free. Then, login and navigate to the UPLOAD tab, where you will find detailed instructions on how to use the tool. If you encounter any issues or have any questions when using PACE, please email us at* [*figures@plos.org*](mailto:figures@plos.org)*. Please note that Supporting Information files do not need this step.*
