# Supplementary material for: Predictors of early initiation of breastfeeding in Indonesia: A population-based cross-sectional survey
Source: PLoS One. 2020 Sep 24;15(9):e0239446. doi: 10.1371/journal.pone.0239446 (PMC7514028; doi:10.1371/journal.pone.0239446)
Supplement: Attachment — Submitted filename: Response to Reviewers .docx [file pone.0239446.s002.docx]

***Review Comments to the Author

Please use the space provided to explain your answers to the questions above. You may also include additional comments for the author, including concerns about dual publication, research ethics, or publication ethics. (Please upload your review as an attachment if it exceeds 20,000 characters)

Reviewer #1: The authors responded appropriately to the reviewer's suggestions, especially in the introduction section. However, although the authors have made several changes throughout the text, the methods, results, and discussion section still need to be carefully reviewed. A more appropriate scientific language is also still required. The general comments below may help the authors to improve the manuscript.

-Review the results presented in the abstract: How do those factors impact on the EIBF?***

**Response: We have revised the abstract. Thank you.**

***-In the dependent variable paragraph, you used EIBF definitions instead of describing how it was measured, and vice versa. The definition of EIBF is different from how it was collected or measured in the population.***

**Response: Thank you. We have added the definition of EIBF in the introduction section.**

***-The description of the independent variables and the results of table 1 and 2 should be presented more succinctly.***

**Response: We have revised it.**

***-This kind of comment is not appropriate in the result section: “Perhaps children whose fathers are not working have working mothers...”***

**Response: Thank you. We have moved the sentence to discussion section.**

***-Table 1 and 2: Check all the variables and make sure the terms you choose are consistent: for example, “Fathers' occupation” or “Husband's occupation”?***

**Response: Thank you for your comments. Father of the infant means husband of the respondent in the survey. For consistency, we have changed husband’s occupation to father’s occupation.**

***-When you say “As mentioned earlier…” Check if you are repeating information.***

**Response: Thank you. We have revised the sentences and checked that there is no repeating information from the earlier paragraph.**

***-Please, be careful with the acronyms used in tables***

**Response: Thank you. We have revised the table, especially in the second table related to Professional assistance during delivery.**

***-For “Mother working status, skin-to-skin contact, and size at birth” results have not been widely discussed.***

**Response: We have added some explanations in the discussion section.**

***-Fathers' occupation (in non-agricultural activities) was not discussed, but it was mentioned as a predictor in this study.***

**Response: Thank you. We have added the discussion related to father’s occupation.**

***Reviewer #2: The authors attended the suggestions and improved the manuscript substantially. However, in my opinion, some minor points should be still addressed before publication:

Abstract:
- Line 21-22: medical level? The methods section describes only three levels.***

**Response: Thank you. We have removed it, because we only discuss individual, household and community levels.**

***Methods:
- In the revised version of the manuscript, the authors used the acronym for delayed initiation of breastfeeding (DIBF) in line 109, but continued using the full name in other sentences (e.g.: line 112). I suggest checking it out.***

**Response: We have revised it as your suggestion. Thank you.**

***- Lines 104-105: To make the text more succinct and avoid repetition, I suggest deleting the sentence: “By definition, these children would be aged between 0 and 23 months at the time of the survey.”***

**Response: We have removed it. Thank you.**

***Results:
- To make this section more concise and easier to understand, I personally suggest the authors to rewrite the second paragraph (lines 223-251). As it contains only descriptive information, statistical significance should not be attributed (e.g. line 238: “Having skin-to-skin contact soon after birth is associated with a higher prevalence of EIBF.”).***

**Response: We have revised it.**

***- Lines 236-237 and 263-264: Sentences should be moved to discussion section.***

**Response: We have moved the sentences to the discussion section.**

***- Table 1: In order to make this table clearer, I suggest deleting the column “Total” (number and %) and line “Total” (1st line) and adding the total number of EIBF and DIBF in the title line [e.g.: Early initiation of breastfeeding (n=3,742) / Delayed initiation of breastfeeding (n=2,874)].***

**Response: Thank you. We have revised Table 1 as suggested.**

***Conclusion:
- I would consider deleting the sentence from lines 418-420 (“This study has been aimed…”) and including a sentence briefly presenting the EIBF predictors found in this study.***

**Response: Thank you. We have revised as suggested.**

***Reviewer #3: The revised manuscript brings a clearer description of the analytical approach. However, I have still questions and comments to further improve the manuscript. A detailed comment is below:
Abstract
Lines 25-27 – Please clarify what prominent means. Are these the factors associated with EIBF? If so, report the OR consistently as reported to C-section.***

**Response: prominent means significant. We have added OR in abstract.**

***Line 29 - Mothers delivering with C-section were less likely to practise EIBF compared to which group. Please include.***

**Response: We have added: “compared to those who have normal vaginal deliveries”.**

***Line 30 - It is not clear in the abstract why skin-to-skin and normal delivery are the strongest factors influencing EIBF. Please report the data in the results to support your conclusions.***

**Response: It is because the adjusted OR of skin-to-skin contact is the highest among all predictors. We have added it in the Results section.**

***Line 31 – replace - early initiation of breastfeeding to EIBF***

**Response: We have replaced it. Thank you.**

***Results
Results should be summarized to highlight the main findings. In its current form it is very long and not informative. Please be careful to not make inferences in the result section. Eg. Lines 236-237, 234-235, etc. Any inferences if appropriate should be placed in the discussion and corroborated with the literature.***

**Response: We have placed these lines in the Discussion section as suggested.**

***Tables
Tables - Please organize your variables in a consistent order. You should organize your variables within the tables following the description given in the methods – under the categories of individual, individual, household and community level variables.***

**Response: We have revised the table as suggested.**

***Table 1 - remove the 100% column. It is unnecessary.***

**Response: We have removed it. Thank you.**

***Table 2 – Please double-check the unadjusted OR for working women it seems incorrect.***

**Response: Thank you. We have checked and revised it based on the data analysis.**

***Conclusions
There were many factors significantly associate with EIBF why authors are issuing recommendations just for skin-to-skin and C-section. Please revise conclusions accordingly specifying the key associated/protective factors that need tackling and whether the counselling strategies should be put in place through hospitals or communities or both?***

**Response: Thank you. We have revised the conclusion.**

**7. PLOS authors have the option to publish the peer review history of their article (**[**what does this mean?**](https://journals.plos.org/plosone/s/editorial-and-peer-review-process#loc-peer-review-history)**). If published, this will include your full peer review and any attached files.


If you choose “no”, your identity will remain anonymous but your review may still be made public.


Do you want your identity to be public for this peer review? For information about this choice, including consent withdrawal, please see our** [**Privacy Policy**](https://www.plos.org/privacy-policy)**.

Reviewer #1: No

Reviewer #2: No

Reviewer #3: No


[NOTE: If reviewer comments were submitted as an attachment file, they will be attached to this email and accessible via the submission site. Please log into your account, locate the manuscript record, and check for the action link "View Attachments". If this link does not appear, there are no attachment files.]

While revising your submission, please upload your figure files to the Preflight Analysis and Conversion Engine (PACE) digital diagnostic tool,** [**https://pacev2.apexcovantage.com/**](https://pacev2.apexcovantage.com/)**. PACE helps ensure that figures meet PLOS requirements. To use PACE, you must first register as a user. Registration is free. Then, login and navigate to the UPLOAD tab, where you will find detailed instructions on how to use the tool. If you encounter any issues or have any questions when using PACE, please email PLOS at** [**figures@plos.org**](mailto:figures@plos.org)**. Please note that Supporting Information files do not need this step.**
