# Supplementary material for: Predictors of early initiation of breastfeeding in Indonesia: A population-based cross-sectional survey
Source: PLoS One. 2020 Sep 24;15(9):e0239446. doi: 10.1371/journal.pone.0239446 (PMC7514028; doi:10.1371/journal.pone.0239446)
Supplement: Attachment — Submitted filename: Response to Reviewer.docx [file pone.0239446.s003.docx]

***Review Comments to the Author***

**Please use the space provided to explain your answers to the questions above. You may also include additional comments for the author, including concerns about dual publication, research ethics, or publication ethics. (Please upload your review as an attachment if it exceeds 20,000 characters)**

**Reviewer #1: The authors followed most of the reviewer's suggestions; however, some points still need to be carefully reviewed. The general comments below may guide the authors:

Abstract:
-Is the confidence interval 95%? Please add this information.**

**Response: Thank you. Yes, it is 95% confidence interval. We have specified “95% CI” in the abstract section, as advised.**

**-The result and conclusion paragraphs repeat information.**

**Response: Thank you. We have revised the conclusion.**

**-Please, note that the abstract exceeds the number of words allowed.**

**Response: Thank you. We have revised the abstract, which has less than 300 words now.**

**Main text:
-The following sentences repeat information: “For more than one child born to a woman in the two years preceding the survey, information pertaining to the most recently born child is analysed in this study.” and “The dependent variable in this study is early initiation of breastfeeding of the woman’s most recent child born in the two years preceding the survey.”**

**Response: Thank you. We have removed the first sentence and kept the second sentence.**

**-In the beginning of the dependent variable section you can use the acronym “EIBF” as you have already mentioned it before in the introduction.
-You do not need to abbreviate EIBF in this section again.**

**Response: Thank you. We have kept the first mention of “Early Initiation of Breastfeeding (EIBF)” in the Introduction and used only the acronym thereafter.**

**-With the following information, please reorganize the dependent variable paragraph (you mixed "definition, measurement and categorization”): 1) Definition of EIBF: “The practice of initiating breastfeeding within the first hour of birth = Early Initiation of Breastfeeding” 2) How was the information on EIBF collected or measured?: “The timing of the initiation of breastfeeding is reported by the women in terms of the number of hours or days after delivery when they started breastfeeding their babies.” 3) Categorization of EIBF for analyses: “The dependent or outcome variable is defined(?) in a binary form, namely EIBF or delayed initiation of breastfeeding (DIBF).” 4) WHO indicator to estimate EIBF: “proportion of infants aged 0-23 months who were put to their mother’s breast within one hour of birth” (WHO. Indicators for assessing infant and young child feeding practices part I: definitions. WHO; 2008.)**

**Response: Thank you. We have reorganized the dependent variable.**

**-You could consider the next example to present the independent variables: “Data were obtained from the hospital records, which included gestational age at delivery (<37 or >=37 weeks), type of delivery (vaginal or caesarean), child’s sex (female or male)”. This way you will have a more succinct paragraph.**

**Response: Thank you for your suggestions. We have revised the independent.**

**-You use child’s size at birth as a proxy for birth weight, but what information was used to classify babies as large or small (small/large for gestational age)? I could not understand this variable.**

**Response: Each woman who had ever given birth was asked the following question with respect to her two most recent births: “When (NAME) was born, was (NAME) very large, larger than average, average, smaller than average, or very small”? (Question 426, Woman’s Questionnaire, Appendix A, Indonesia Demographic and Health Survey 2017, Final Report p.475). This was done especially when the birth took place at home where there was no facility to weigh the child at birth** (National Population and Family Planning Board, Statistics Indonesia, Ministry of Health, & ICF, 2018, p.160)**. The child’s size at birth was thus recorded according to the woman’s perception of her child’s size at birth. The woman’s response of the child being “very small” or “smaller than average,” was taken to be a proxy for Low Birth Weight.**

**-Thanks for the description of the wealth index, but please summarize the paragraph. It is important to mention briefly where it comes from and its reference.**

**Response: Thank you. We have mentioned the source from the DHS Program.**

**-The ‘svy’ command explanation is not necessary.**

**Response: Thank you. We have removed it in the method section.**

**-The main characteristics of the study population should be presented succinctly (lines 226 to 261).**

**Response: Thank you, we have revised it.**

**-This kind of comment is not appropriate in the result section: “Women who give birth to small sized babies are probably themselves undernourished compared to the women who give birth to average or large sized babies.”**

**Response: Thank you. We have removed it.**

**-What is the reference for the following information? “…although it is higher than that in Vietnam (27%), Thailand 312 (40%) and Lao People’s Democratic Republic (50%)”**

**Response: The data from the UNICEF. We have added the reference.**

**-The information presented from line 328 to 343 I do not think is necessary.**

**Response: Thank you. However, we think that this part is important for providing clarifications about those predictors that have no association with EIBF in Indonesia.**

**-“In terms of education however, studies in other countries have shown...” which countries?**

**Response: in Ethiopia and Malawi** (Liben & Yesuf, 2016; Nkoka, Ntenda, Kanje, Milanzi, & Arora, 2019)**.**

**-“This apparent anomaly may be explained by…” what do you mean by anomaly?**

**Response: By “anomaly” we mean “difference or “irregularity”. We have re-written the sentence and replaced the word “anomaly’ with the word “difference”**

**- Please, avoid “(table 1)” in discussion section.**

**Response: Thank you. We have removed “table 1” in discussion section.**

**-What does “[12 Figure 3.3]” mean? Line 357.**

**Response: We have removed it. Thank you.**

**-Is this information related to EIBF? “working women might be concerned about their professional and body image, so they might consider breastfeeding an obstacle to return to their physical shape of the time before they became pregnant [18,24].”**

**Response: It might be related. Working women who are concerned about their body image might choose not to breastfeed their babies, or choose to curtail their breastfeeding period, thus adversely affecting EIBF and exclusive breastfeeding practices. We have provided references to the sources of such a statement.

-This information is repeated in the text:
“the IDHS 2017 data were collected by using a two-stage stratified sampling.”**

**Response: Thank you, we have removed the repetition.**

**“As mentioned earlier, the data used in this research were collected at the 2017 Indonesia Demographic and Health Survey.”**

**Response: Thank you, we have removed the repetition.**

**“…had at least one live birth during the two years preceding the survey and who breastfed their most recently born child.”**

**Response: Thank you, we have removed the repetition.**

**“confidence interval (CI)”**

**Response: Thank you, we have removed the repetition.**

**“the Indonesia Demographic and Health Survey (IDHS)”**

**Response: Thank you, we have kept it where it is mentioned for the first time in the main text, but removed the repetition thereafter.**

**“(proxy for birth weight)”**

**Response: Thank you, we have removed the repetition.**

**“the occupation of women’s husbands (father’s occupation) is found to be associated with EIBF in a manner that is different from the association of EIBF with women’s occupation.” (similar words)**

**Response: Thank you, we have removed it due to repetition. It has been explained in the next sentence.**

**“as mentioned earlier, EIBF has benefits for the health of both mothers and infants”**

**Response: Thank you, we have removed the repetition.**

**“early initiation of breastfeeding (EIBF)”**

**Response: Thank you, we have removed the repetition.**

**“(see Table 1)” = “The categories of each variable are given in Table 1”.**

**Response: Thank you, we have revised the sentence as you have suggested.**

**Reviewer #2: The authors made several improvements in the text. The points I raised in response to the last submission of this manuscript have been sufficiently addressed. I consider that the article is now suitable for publication in this Journal.**

**Response: Thank you.**

**7. PLOS authors have the option to publish the peer review history of their article (**[**what does this mean?**](https://journals.plos.org/plosone/s/editorial-and-peer-review-process#loc-peer-review-history)**). If published, this will include your full peer review and any attached files.


If you choose “no”, your identity will remain anonymous but your review may still be made public.


Do you want your identity to be public for this peer review? For information about this choice, including consent withdrawal, please see our** [**Privacy Policy**](https://www.plos.org/privacy-policy)**.**

**Reviewer #1: No**

**Reviewer #2: No**

**[NOTE: If reviewer comments were submitted as an attachment file, they will be attached to this email and accessible via the submission site. Please log into your account, locate the manuscript record, and check for the action link "View Attachments". If this link does not appear, there are no attachment files.]

While revising your submission, please upload your figure files to the Preflight Analysis and Conversion Engine (PACE) digital diagnostic tool,** [**https://pacev2.apexcovantage.com/**](https://pacev2.apexcovantage.com/)**. PACE helps ensure that figures meet PLOS requirements. To use PACE, you must first register as a user. Registration is free. Then, login and navigate to the UPLOAD tab, where you will find detailed instructions on how to use the tool. If you encounter any issues or have any questions when using PACE, please email PLOS at** [**figures@plos.org**](mailto:figures@plos.org)**. Please note that Supporting Information files do not need this step.**

**References:**

Liben, M. L., & Yesuf, E. M. (2016). Determinants of early initiation of breastfeeding in Amibara district, Northeastern Ethiopia: A community based cross-sectional study. *International Breastfeeding Journal*, *11*(1), 1–7. https://doi.org/10.1186/s13006-016-0067-8

National Population and Family Planning Board, Statistics Indonesia, Ministry of Health, & ICF. (2018). *Indonesia Demographic and Health Survey 2017*. Jakarta.

Nkoka, O., Ntenda, P. A. M., Kanje, V., Milanzi, E. B., & Arora, A. (2019). Determinants of timely initiation of breast milk and exclusive breastfeeding in Malawi: A population-based cross-sectional study. *International Breastfeeding Journal*, *14*(1), 1–9. https://doi.org/10.1186/s13006-019-0232-y
